# White Matter Microstructural Alterations Mediate the Association between Polygenic Risk for Alzheimer’s Disease and Cognitive Performance in Clinically Asymptomatic Adults

**DOI:** 10.2463/mrms.mp.2025-0182

**Published:** 2026-02-06

**Authors:** Rui Zou, Kaito Takabayashi, Christina Andica, Wataru Uchida, Takafumi Kitagawa, Koyo Mizuta, Akifumi Hagiwara, Junko Kikuta, Shigeki Aoki, Koji Kamagata

**Affiliations:** 1 Department of Radiology, Juntendo University Graduate School of Medicine, Tokyo, Japan; 2 Department of Data Science, Juntendo University Graduate School of Medicine, Tokyo, Japan; 3 Faculty of Health Data Science, Juntendo University, Urayasu, Chiba, Japan; 4 Graduate School of Health Data Science, Juntendo University, Urayasu, Chiba, Japan

**Keywords:** cognitive function, neurite orientation dispersion and density imaging, polygenic risk score of Alzheimer’s disease, UK Biobank, white matter microstructure

## Abstract

**Purpose:**

Alzheimer’s disease (AD) is a progressive neurodegenerative disorder influenced by genetic factors, with a long preclinical phase characterized by subtle alterations in brain microstructure. Although the apolipoprotein E ε4 allele is a well-established genetic risk factor, AD is increasingly recognized as a polygenic condition. However, how polygenic risk manifests in white matter (WM) microstructure and cognition remains unclear. This study aims to investigate the associations between AD polygenic risk scores (ADPRS), WM microstructure, and cognitive performance in clinically asymptomatic adults.

**Methods:**

Data from 36400 individuals (aged 45–83 years) in the UK Biobank were analyzed. Diffusion tensor imaging and neurite orientation dispersion and density imaging metrics were extracted from 48 WM tracts. General linear models were used to examine associations between ADPRS, WM integrity, and 10 cognitive measures. Mediation analyses were conducted to test whether WM microstructure mediated the relationship between ADPRS and cognitive performance.

**Results:**

Higher ADPRS was significantly associated with reduced fractional anisotropy and intracellular volume fraction, and with increased mean, axial, and radial diffusivity, as well as isotropic volume fraction across limbic and association fibers, particularly in the cingulum hippocampus, fornix, posterior thalamic radiation, and superior fronto-occipital fasciculus. Higher ADPRS was also associated with poorer cognitive performance, most prominently on tests of executive function (Trail Making Test B), episodic memory (Paired Associate Learning), and processing speed (Symbol Digit Substitution). Mediation analysis revealed that WM microstructural alterations, especially increased radial diffusivity and reduced fractional anisotropy in the posterior thalamic radiation, partially mediated the association between ADPRS and cognitive performance, accounting for up to 4.88% of the total effect.

**Conclusion:**

ADPRS is linked to selective WM microstructural alterations and subtle cognitive difference in clinically asymptomatic adults. WM microstructural changes partially mediate the association between ADPRS and cognitive performance.

## Introduction

Alzheimer’s disease (AD) is a progressive neurodegenerative disorder influenced by genetic factors, characterized by a prolonged preclinical phase lasting approximately 15–20 years and complex pathological alterations including *β*-amyloid accumulation, tauopathy, synaptic loss, and neuroinflammation.^1^^–^^4^ With over 416 million people estimated to be living across the AD continuum, including preclinical AD, prodromal AD and AD dementia, and a prevalence that rises sharply with age, affecting approximately 22% of individuals aged 50 and above, AD poses a growing global health challenge.^5^ Although recent advances in treatment have modestly slowed the progression of symptoms, no curative treatment is available. Thus, identifying early pathological alterations and the genetic mechanisms contributing to AD onset is crucial for developing early detection and effective preventive strategies.

Neuroimaging has emerged as a critical tool for tracking preclinical AD-related microstructural alterations including neuronal and synaptic atrophy, as well as white matter (WM) microstructure alterations.^6^^–^^8^ Among imaging modalities, diffusion MRI (dMRI), including diffusion tensor imaging (DTI) and neurite orientation dispersion and density imaging (NODDI), enables non-invasive assessment of WM microstructure *in vivo*.^9^^–^^11^ Compared to conventional DTI metrics like fractional anisotropy (FA) and mean diffusivity (MD), the NODDI model offers biologically interpretable parameters such as intracellular volume fraction (ICVF) and isotropic volume fraction (ISOVF), which reflect neurite density and extracellular free water, respectively.^12^ These parameters are especially sensitive for detecting early AD-related processes, including axonal degeneration and neuroinflammation, and may serve as valuable biomarkers of the disease’s preclinical phase.^6^^,^^9^^,^^13^^,^^14^

Genetic factors also play a significant role in AD progression. The apolipoprotein E (*APOE*) ε4 allele is the most well-established common risk factor for late-onset AD, conferring a 3-fold increased risk and an earlier age of onset.^15^^–^^17^ Beyond *APOE*, AD is increasingly recognized as a polygenic disorder, with susceptibility shaped by the cumulative effect of numerous common and rare genetic variants. While rare mutations in genes such as *APP*, *PSEN1*, and *PSEN2* contribute to early-onset familial AD,^18^^,^^19^ genome-wide association studies (GWAS) have identified over 75 loci associated with late-onset AD.^20^ Polygenic risk scores (PRS), which aggregate the effects of these variants into a single quantitative metric, provide a powerful approach to quantify genetic liability and stratify individuals according to their risk.^21^^–^^23^

Recent advances have enabled the integration of PRS of AD (ADPRS) with neuroimaging data, offering new insights into how genetic susceptibility manifests in brain structure of AD prior to clinical onset.^24^^,^^25^ Previous studies have shown that individuals with higher ADPRS tend to exhibit reduced gray matter (GM) volume, particularly in the hippocampus,^26^^–^^28^ along with alterations in WM microstructure, such as increased MD and decreased FA across widespread WM tracts. Although DTI-based measures such as FA and MD are sensitive to microstructural changes, they lack specificity in distinguishing the underlying pathological processes such as axonal degeneration, demyelination, or extracellular fluid accumulation. To overcome these limitations, we employed NODDI, which provides biologically informative diffusion metrics that enable more precise characterization of WM microstructure, including neurite density (ICVF) and extracellular free water (ISOVF). Using these biophysically informed diffusion metrics, we aim to elucidate how ADPRS is associated with specific WM microstructural alterations and whether these changes contribute to early cognitive vulnerabilities in clinically asymptomatic individuals.

To address these gaps, the present study investigated the relationships between ADPRS, WM microstructure, and cognitive performance in a large cohort of clinically asymptomatic adults from the UK Biobank (UKB). By integrating 10 cognitive function assessments and advanced dMRI metrics from both DTI and NODDI across 48 WM tracts, we aimed to (1) determine whether higher ADPRS is associated with subtle but spatially specific WM alterations and cognitive deficits and (2) examine whether selective WM microstructure metrics mediate the relationship between genetic risk and cognitive functions. This integrative approach offers a novel window into the early neurobiological mechanisms of AD and highlights the potential utility of combining polygenic profiling with neuroimaging to stratify risk and inform preventive strategies in preclinical AD.

## Materials and Methods

### Participants

Data were drawn from the UKB, an open-access large-scale prospective cohort study with phenotypic, genotypic, and neuroimaging data comprising over 500000 participants recruited between 2006 and 2011 across the United Kingdom.^29^ The UKB study has received ethical approval from the National Health Service North West Centre for Research Ethics Committee (reference: 16/NW/0274), and the present study was conducted based on application 135200.

A total of 36400 individuals aged 45–83 (mean age = 64.06 ± 7.71 years) with available T1-weighted image (T1WI), dMRI, and PRS for AD were included.^30^^–^^32^ To minimize the potential confounding from neurological disorders, we excluded individuals with diagnoses of dementia, AD, mild cognitive impairment (MCI), brain injury, brain tumors, or other central nervous system disorders as classified by the International Classification of Diseases, 10th Revision (ICD-10). The full list of ICD-10 codes for exclusion is provided in Table S1. Accordingly, the final analytic sample consisted of clinically asymptomatic individuals, defined as participants without clinically diagnosed dementia, AD, MCI, or major neurological disorders at the time of assessment.

### MRI acquisition and processing

The MRI data were acquired using 3 Tesla Skyra system (Siemens Healthineers, Erlangen, Germany) equipped with a standard Siemens 32-channel receive head coil as part of the UKB imaging protocol.^32^ Acquisition parameters for the T1WI and dMRI sequence are provided in the Table S2.

After preprocessing, mean values of FA, MD, axial diffusivity (AxD), radial diffusivity (RD), ICVF, ISOVF, and orientation dispersion index (OD) for each participant were extracted from 48 WM tracts defined by the Johns Hopkins University ICBM-DTI-81 atlas.^33^^,^^34^ Further details of data processing on the UKB imaging pipeline are available at: https://www.ukbiobank.ac.uk/enable-your-research/about-our-data/imaging-data.

### PRS calculation

The ADPRS used in this study were generated by UKB (field ID: 26206) based on a fixed-effect inverse variance-weighted meta-analysis of 2 large-scale, publicly available GWAS datasets that excluded any UKB participants. The combined GWAS data included 42588 AD cases and 573011 controls, with overlapping samples between the 2 source datasets accounted for during meta-analysis.^30^^,^^35^

Variants included in the PRS model were filtered for quality and consistency across studies, and PRS weights were generated using a Bayesian approach applied to the meta-analyzed summary statistics. The individual PRS values were calculated as the sum over all variants of the posterior effect size multiplied by the individual’s allele dosage. Because no UKB genetic data were used during PRS construction, the Standard ADPRS are independent of the UKB cohort used for analysis.^30^^,^^35^

### Cognitive assessment

Participants in the UKB completed 10 cognitive tests either in assessment centers or during online follow-up. The selected tests included: trail making test A (TMT-A), trail making test B (TMT-B), reaction time (RT), numeric memory (NM), prospective memory (PM), fluid intelligence score (FIS), matrix pattern completion (MPC), symbol digit substitution (SDS), picture vocabulary (PV), and paired associate learning (PAL). Descriptions of each cognitive test and their scoring metrics are provided in Table S3.^36^ Notably, lower scores indicate better cognitive function on TMT-A, TMT-B and RT, whereas higher scores indicate better cognitive function on the other cognitive tests.

### Statistical analysis

All statistical analyses were performed in R (version 4.4.0). General linear model (GLM) was employed to evaluate the associations between ADPRS and dMRI-derived WM metrics, as well as between ADPRS and cognitive function scores. Covariates included age, age^2^, sex, intracranial volume (ICV), and the first 10 genetic principal components (PCs) to control for potential confounding effects. All variables, including dMRI metrics, cognitive function scores, ADPRS, and covariates, were standardized using Z-score transformation (mean = 0, standard deviation = 1). In addition to analyses using continuous ADPRS values, subgroup comparisons were conducted between individuals in the top 25% and bottom 25% of the ADPRS distribution. GLMs were applied for each comparison, adjusting for the same covariates. Multiple comparison correction was applied using the false discovery rate (FDR) method with a significance threshold of FDR-adjusted *P* < 0.05. To assess whether WM microstructure alterations mediate the relationship between ADPRS and cognitive outcomes, we performed mediation analysis using PROCESS v4.3 for R, with ADPRS as the independent variable, 336 combinations of dMRI-derived metrics (7 metrics × 48 WM tracts) as the mediators, and 10 cognitive test scores as the dependent variables.

### Data and code availability

All data used in this study were obtained from the UKB (https://www.ukbiobank.ac.uk/). The specific imaging, ADPRS, and cognitive variables used are listed in Table S4.

## Results

### Characteristics of participants

A total of 36400 UKB participants were initially included. Due to missing data in imaging or cognitive measures, the final analytic sample size varied across analyses. Demographic and cognitive characteristics stratified by ADPRS quartiles are summarized in Table 1. Participants were divided into 4 groups based on ADPRS quartiles. While age, sex, ICV, and 10 cognitive test scores were broadly comparable across groups, significant differences were observed in ADPRS, age, and selected cognitive measures such as TMT-B, PAL, FIS, SDS, and MPC (Table 1).

### ADPRS is associated with dMRI metrics in selective brain regions

**Fig. 1:**
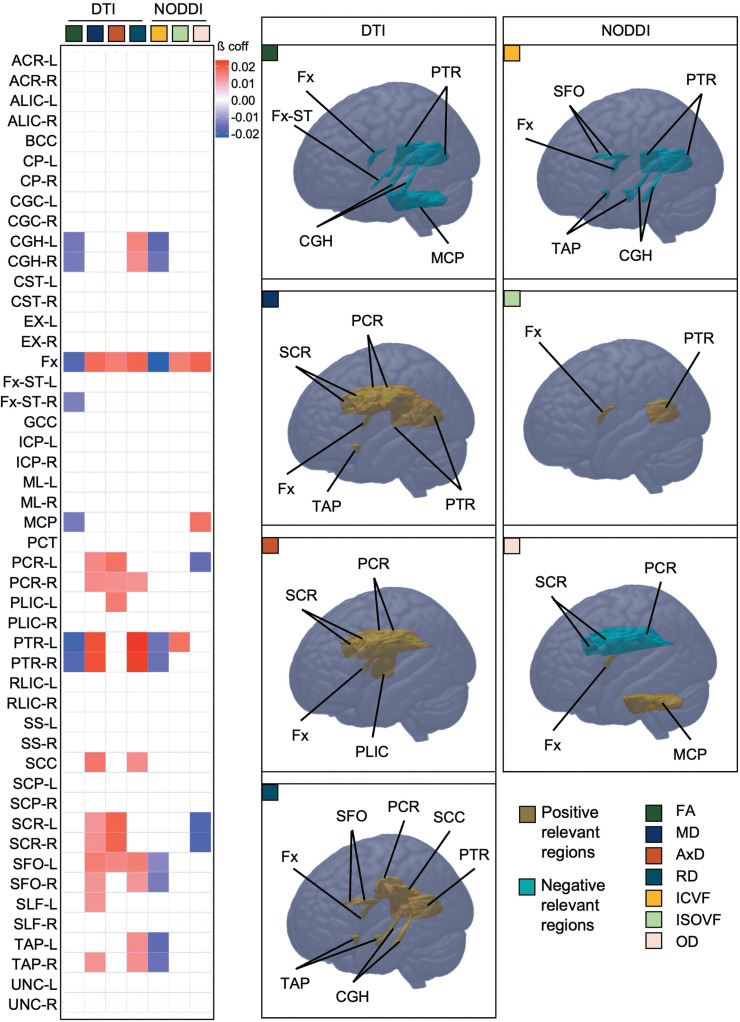
Global associations patterns between ADPRS and WM microstructure across multiple dMRI metrics. The left panel presents a heatmap showing standardized *β* coefficients from general linear models testing the association between ADPRS and dMRI-derived measures across 48 WM tracts in 36400 cognitively normal adults. Metrics include DTI measures—FA, MD, AxD, and RD—and NODDI measures—ICVF, ISOVF, and OD. Warm colors (red) indicate positive associations, while cool colors (blue) indicate negative associations. All models were adjusted for age, sex, age^2^, ICV, and the first 10 genetic principal components. Results were corrected for multiple comparisons using FDR (FDR < 0.05). The right panel displays 3D tract renderings of WM regions showing significant associations with ADPRS for each dMRI metric. DTI results are shown in the left column of the 3D renderings, and NODDI results in the right column. Highlighted tracts include the Fx, CGH, PTR, SFO, TAP, PCR, and MCP. Color coding reflects the direction of association: gold indicates positive and cyan indicates negative effects.ADPRS, Alzheimer’s disease polygenic risk scores; AxD, axial diffusivity; CGH, cingulum hippocampal part; dMRI, diffusion MRI; DTI, diffusion tensor imaging; FA, fractional anisotropy; FDR, false discovery rate; Fx, fornix; ICV, intracranial volume; ICVF, intracellular volume fraction; ISOVF, isotropic volume fraction; MCP, middle cerebellar peduncle; MD, mean diffusivity; NODDI, neurite orientation dispersion and density imaging; OD, orientation dispersion; PCR, posterior corona radiata; PTR, posterior thalamic radiation; RD, radial diffusivity; SFO, superior fronto-occipital fasciculus; TAP, tapetum; WM, white matter.

We examined the association between ADPRS and dMRI-derived metrics across 48 WM tracts. After adjusting for covariates and applying FDR correction at *P* < 0.05, 18 regions showed significant associations (Fig. 1). Higher ADPRS was significantly associated with reduced FA and ICVF, as well as increased MD, AxD, RD, and ISOVF in limbic and association fibers, including the cingulum hippocampus (CGH), fornix (Fx), posterior corona radiata (PCR), superior corona radiata (SCR), posterior thalamic radiation (PTR), superior fronto occipital fasciculus (SFO), and tapetum (TAP). Notably, ICVF exhibited significant negative associations with ADPRS in the CGH (Left [L]: *β* = −0.0175, *P* = 0.0156; Right [R]: *β* = −0.0149, *P* = 0.0225), Fx (*β* = −0.0220, *P* = 0.0007), PTR (L: *β* = −0.0151, *P* = 0.0202; R: *β* = −0.0154, *P* = 0.0202), SFO (L: *β* = −0.0121, *P* = 0.0451; R: *β* = −0.0138, *P* = 0.0202), and TAP (L: *β* = −0.0164, *P* = 0.0156; R: *β* = −0.0153, *P* = 0.0202); while ISOVF demonstrated significant positive associations with ADPRS in the Fx (*β* = 0.0155, *P* = 0.0144) and left of PTR (*β* = 0.0170, *P* = 0.0249) (Fig. 1).

### Higher ADPRS is associated with poorer cognitive function

Higher ADPRS was also significantly associated with poorer cognitive performance (Table 2). Specifically, increased ADPRS was related to longer completion times on both TMT-A (*β* = 0.0128, *P* = 0.0426) and TMT-B (*β* = 0.0266, *P* < 0.0001), indicating slower processing speed and executive function. In addition, significant negative associations were found between ADPRS and performance on several other cognitive domains, including PM (*β* = −0.0199, *P* = 0.0003), PAL (*β* = −0.0338, *P* < 0.0001), FIS (*β* = −0.0203, *P* = 0.0003), SDS (*β* = −0.0370, *P* < 0.0001), MPC (*β* = −0.0271, *P* < 0.0001). For NM, the association was marginal (*β* = −0.0117, *P* = 0.0682), and no significant associations were observed for RT or PV (*P* > 0.1). These findings suggest that ADPRS is linked to subtle but measurable impairments in multiple cognitive domains, particularly those related to executive function, memory, and processing speed.

### Stratified analysis reveals PRS-associated WM vulnerability

**Fig. 2:**
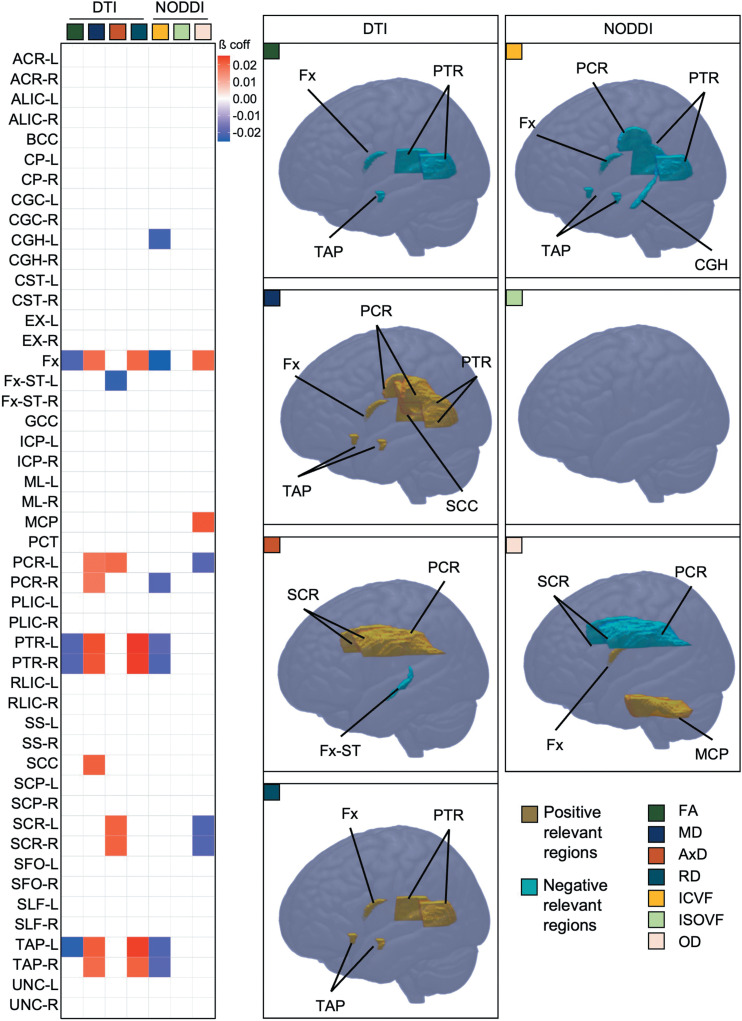
Group comparisons of WM microstructure between high and low ADPRS groups. Comparison of dMRI-derived WM integrity between individuals in the top (≥ 75th percentile) and bottom (≤ 25th percentile) quartiles of ADPRS. The heatmap (left) shows group-level differences in FA, MD, AxD, RD, ICVF, ISOVF, and OD across 48 WM tracts. Metrics include DTI measures—FA, MD, AxD, and RD—and NODDI measures—ICVF, ISOVF, and OD. Warm colors (red) indicate positive associations, while cool colors (blue) indicate negative associations. All models were adjusted for age, sex, age^2^, ICV, and the first 10 genetic principal components. Results were corrected for multiple comparisons using FDR (FDR < 0.05). 3D tract renderings (right) illustrate spatial patterns of group differences with ADPRS for each dMRI metric. DTI results are shown in the left column of the 3D renderings, and NODDI results in the right column. Highlighted tracts include the Fx, CGH, PTR, TAP, PCR, and MCP. Color coding reflects the direction of association: gold indicates positive and cyan indicates negative effects.ADPRS, Alzheimer’s disease polygenic risk scores; AxD, axial diffusivity; CGH, cingulum hippocampal part; dMRI, diffusion MRI; DTI, diffusion tensor imaging; FA, fractional anisotropy; FDR, false discovery rate; Fx, fornix; ICV, intracranial volume; ICVF, intracellular volume fraction; ISOVF, isotropic volume fraction; MCP, middle cerebellar peduncle; MD, mean diffusivity; NODDI, neurite orientation dispersion and density imaging; OD, orientation dispersion; PCR, posterior corona radiata; PTR, posterior thalamic radiation; RD, radial diffusivity; SFO, superior fronto-occipital fasciculus; TAP, tapetum; WM, white matter.

**Fig. 3:**
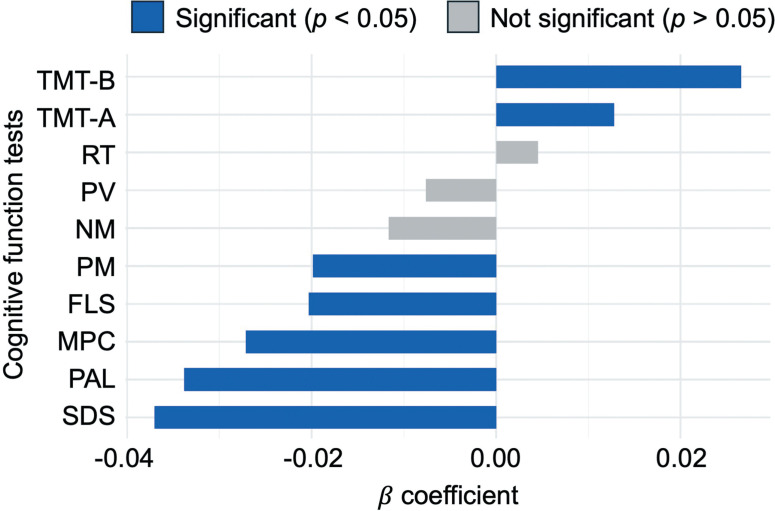
Group comparisons of 10 neuropsychological tests between high and low ADPRS groups. Comparison of 10 neuropsychological tests between individuals in the top (≥ 75th percentile) and bottom (≤ 25th percentile) quartiles of ADPRS. Ten cognitive function tests include TMT-A, TMT-B, RT, NM, PM, FIS, MPC, SDS, PV, and PAL. Lower scores indicate better cognitive function on TMT-A, TMT-B and RT, whereas higher scores indicate better cognitive function on the other cognitive tests. Negative β coefficients indicate the values in high ADPRS group are lower than that in low ADPRS group. Models were adjusted for age, age^2^, sex, educational attainment, intracranial volume, and the first 10 genetic principal components. Significant associations (FDR-corrected *P* < 0.05) are shown in blue, while nonsignificant associations are shown in gray.ADPRS, Alzheimer’s disease polygenic risk scores; FIS, fluid intelligence score; FDR, false discovery rate; MPC, matrix pattern completion; NM, numeric memory; PAL, paired associate learning; PM, prospective memory; PV, picture vocabulary; RT, reaction time; SDS, symbol digit substitution; TMT-A, trail making test A; TMT-B, trail making test B.

To further elucidate the impact of ADPRS on brain WM integrity, we compared individuals in the high (≥ 75th percentile) and low (≤ 25th percentile) quartiles of ADPRS. As shown in Fig. 2, compared to the low ADPRS group, individuals in the high ADPRS group exhibited significant alterations in several WM microstructure metrics, including reduced FA and ICVF and increased MD, AxD, and RD in regions such as the Fx, SCR, PTR, and TAP, suggesting potential demyelination, axonal degeneration, and elevated extracellular water. Moreover, compared to low ADPRS group, cognitive performance was also significantly poorer in the high ADPRS group, particularly in the SDS (*β *= −0.0451, *P* < 0.0001), PAL (*β *= −0.0411, *P* < 0.0001) and MPC (*β *= −0.0337, *P* = 0.0002) (Fig. 3).

**Fig. 4:**
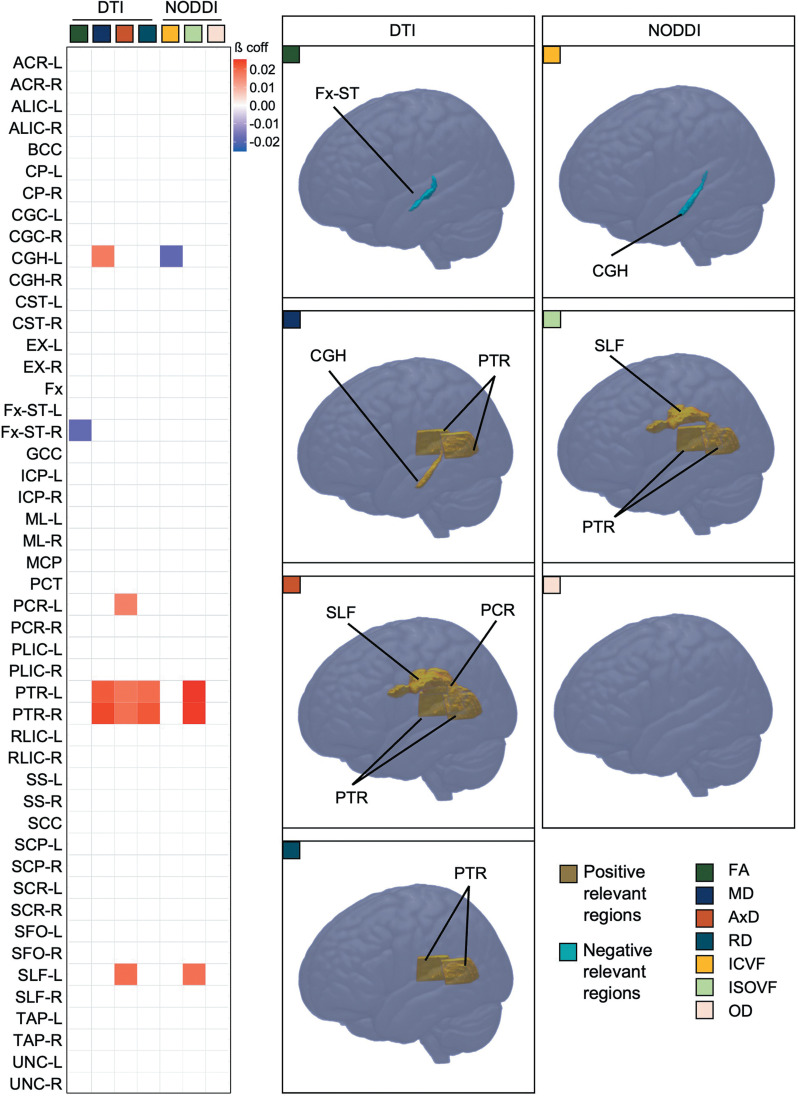
Tract-specific associations between ADPRS and WM microstructure in the high ADPRS group. Results from within-group GLM analyses showing associations between ADPRS and dMRI-derived WM microstructure metrics in individuals within the top ADPRS quartile (≥ 75th percentile). The heatmap (left) shows group-level differences in FA, MD, AxD, RD, ICVF, ISOVF, and OD across 48 WM tracts. Metrics include DTI measures—FA, MD, AxD, and RD—and NODDI measures—ICVF, ISOVF, and OD. Warm colors (red) indicate positive associations, while cool colors (blue) indicate negative associations. All models were adjusted for age, sex, age^2^, ICV, and the first 10 genetic principal components. Results were corrected for multiple comparisons using FDR (FDR < 0.05). 3D tract renderings (right) illustrate spatial patterns of group differences with ADPRS for each dMRI metric. DTI results are shown in the left column of the 3D renderings, and NODDI results in the right column. Highlighted tracts include the CGH, PTR and PCR. Color coding reflects the direction of association: gold indicates positive and cyan indicates negative effects.ADPRS, Alzheimer’s disease polygenic risk scores; AxD, axial diffusivity; CGH, cingulum hippocampal part; dMRI, diffusion MRI; DTI, diffusion tensor imaging; FA, fractional anisotropy; FDR, false discovery rate; Fx, fornix; GLM, general linear model; ICV, intracranial volume; ICVF, intracellular volume fraction; ISOVF, isotropic volume fraction; MCP, middle cerebellar peduncle; MD, mean diffusivity; NODDI, neurite orientation dispersion and density imaging; OD, orientation dispersion; PCR, posterior corona radiata; PTR, posterior thalamic radiation; RD, radial diffusivity; SFO, superior fronto-occipital fasciculus; TAP, tapetum; WM, white matter.

**Fig. 5:**
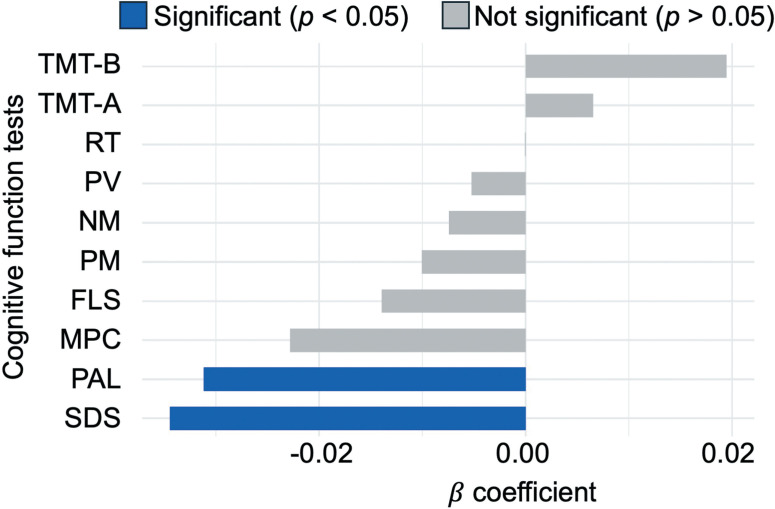
Associations between ADPRS and cognitive performance across 10 neuropsychological tests in high ADPRS group. Bar plot showing standardized β coefficients from general linear models testing the association between ADPRS and performance on 10 cognitive function tests in individuals in the top (≥ 75th percentile) of ADPRS. Ten cognitive function tests include TMT-A, TMT-B, RT, NM, PM, FIS, MPC, SDS, PV, and PAL. Lower scores indicate better cognitive function on TMT-A, TMT-B and RT, whereas higher scores indicate better cognitive function on the other cognitive tests. Negative β coefficients indicate the negative association between ADPRS and the indicated cognitive tests in high ADPRS group. Models were adjusted for age, age^2^, sex, intracranial volume, and the first 10 genetic principal components. Significant associations (FDR-corrected *P* < 0.05) are shown in blue, while nonsignificant associations are shown in gray.ADPRS, Alzheimer’s disease polygenic risk scores; FDR, false discovery rate; FIS, fluid intelligence score; MPC, matrix pattern completion; NM, numeric memory; PAL, paired associate learning; PM, prospective memory; PV, picture vocabulary; RT, reaction time; SDS, symbol digit substitution; TMT-A, trail making test A; TMT-B, trail making test B.

Within-group GLM analysis further revealed that associations between ADPRS, dMRI metrics, and cognitive performance were predominantly observed in the high ADPRS group. For example, lower ICVF was observed in the left CGH (*β* = −0.0336, *P* = 0.0467), and higher ISOVF was found in the bilateral PTR (L: *β* = 0.0454, *P* = 0.0003; R: *β* = 0.0450, *P* = 0.0003) and left superior longitudinal fasciculus (SLF) (*β* = 0.0334, *P* = 0.0196) (Fig. 4). In contrast, no significant associations were identified in the low ADPRS group, highlighting a threshold-like relationship between genetic risk and brain vulnerability. Similarly, significant negative associations between ADPRS and cognitive tests were found for PM (*β* = −0.0312, *P* = 0.0166) and SDS (*β* = −0.0345, *P* = 0.0166) in high ADPRS group (Fig. 5), while no significant associations were detected in the low ADPRS group.

### dMRI metrics mediate the relationship between ADPRS and cognitive function

**Fig. 6:**
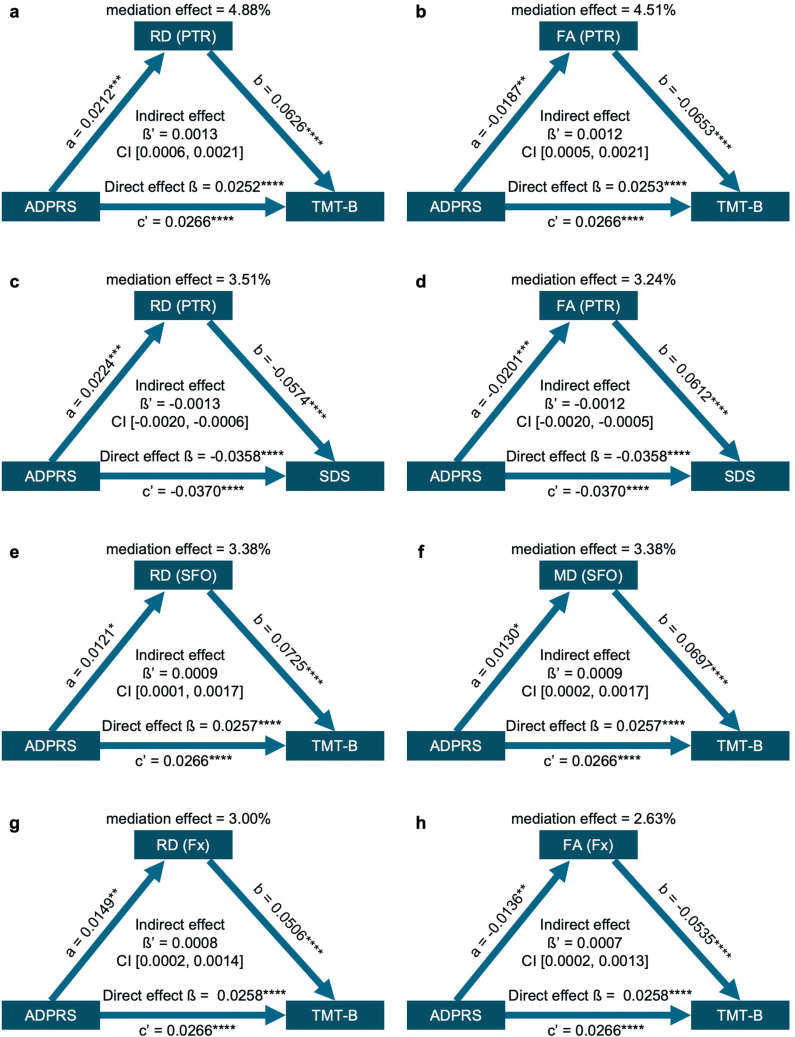
Mediation models illustrating the indirect effects of WM microstructure on the relationship between ADPRS and cognitive performance. Path diagrams (**a**–**h**) show significant mediation effects of dMRI-derived WM metrics on the association between ADPRS and performance on cognitive tasks. Mediation models were adjusted for age, age^2^, sex, intracranial volume, and the first 10 genetic principal components. Indirect effects (a × b paths), direct effects (c′), and total effects (c) are presented with standardized *β* coefficients and 95% CIs. (**a**) RD in the PTR mediates the effect of ADPRS on TMT-B performance (4.88% of total effect). (**b**) FA in the PTR mediates the effect of ADPRS on TMT-B (4.51%). (**c**) RD in the PTR mediates the effect of ADPRS on SDS (3.51%). (**d**) FA in the PTR mediates the effect of ADPRS on SDS (3.24%). (**e**) RD in the SFO mediates the effect of ADPRS on TMT-B (3.38%). (**f**) MD in the SFO mediates the effect of ADPRS on TMT-B (3.38%). (**g**) RD in the Fx mediates the effect of ADPRS on TMT-B (3.00%). (**h**) FA in the Fx mediates the effect of ADPRS on TMT-B (2.63%). *P < 0.05, **P < 0.01, ***P < 0.001, ****P < 0.0001.ADPRS, Alzheimer’s disease polygenic risk scores; CIs, confidence intervals; dMRI, diffusion MRI; FA, fractional anisotropy; Fx, fornix; MD, mean diffusivity; PTR, posterior thalamic radiation; RD, radial diffusivity; SDS, symbol digit substitution; SFO, superior fronto-occipital fasciculus; TMT-B, trail making test B; WM, white matter.

To investigate whether WM microstructure alterations mediate the relationship between ADPRS and cognitive performance, we conducted mediation analysis using dMRI-derived WM metrics as potential mediators. Although the proportion of mediated effects was generally modest (ranging from 0.81% to 4.88%; Table S5), several consistent and biologically plausible patterns emerged. Specifically, the PTR was the most prominent mediator, particularly for executive function, as assessed by the TMT-B task. In the left and right PTR, RD exhibited the strongest mediation effects (both 4.88%; Fig. 6a and Table S5), followed by FA (left: 4.51%; right: 4.13%; Fig. 6b and Table S5) and MD (3.00%; Table S5). The PTR also mediated the effects of ADPRS on SDS, MPC, and PAL performance through multiple dMRI metrics, including RD (3.51%; Fig. 6c) and FA (3.24%; Fig. 6d).

The SFO was another major tract involved in mediating the ADPRS effect on TMT-B. Significant indirect effects were observed for RD and MD (both 3.38%; Fig. 6e, f), as well as ISOVF and AxD (3.00% and 2.63% respectively; Table S5) in the left SFO. FA and ICVF in bilateral SFO also showed indirect effects, though of smaller magnitude (Table S5). The Fx showed consistent but relatively weaker mediation effects on TMT-B. We observed that multiple dMRI metrics, including RD (3.00%; Fig. 6g), FA (2.63%; Fig. 6h), MD, AxD, ICVF, and OD (2.63%, 2.25%, 2.25% and 1.87% respectively; Table S5), contributed to indirect pathways linking ADPRS to performance on TMT-B. Additional effects were found for ICVF in the TAP and ISOVF in the splenium of corpus callosum.

## Discussion

Recent advances in genetics and neuroimaging have made it possible to examine how inherited susceptibility to AD manifests in the brain prior to clinical onset.^25^ PRS, which capture the cumulative contribution of many common genetic variants, have emerged as a promising tool for quantifying inherited susceptibility to late-onset AD.^37^ However, a major challenge remains in translating this probabilistic risk into observable neurobiological alterations. Building on this framework, the present study provides several novel insights into the neurobiological consequences of ADPRS. First, by integrating conventional DTI metrics with biophysically informed NODDI parameters, we show that these alterations are consistent with biologically interpretable microstructural features, including reduced neurite density and increased extracellular free water. Second, we demonstrate that tract-specific WM microstructural alterations partially mediate the association between ADPRS and cognitive performance, supporting WM integrity as an intermediate pathway linking genetic susceptibility to early cognitive vulnerability. Third, using tract-level diffusion analyses in a very large cohort of clinically asymptomatic adults, we demonstrate that ADPRS is associated with spatially selective WM microstructural differences, rather than uniform or global WM alterations.

Specifically, individuals with elevated ADPRS exhibited reduced ICVF and FA, together with increased MD, RD, and AxD in limbic and association tracts such as the CGH, Fx, PTR, and SFO, which are known to be vulnerable in the earliest stages of AD^9^^,^^38^ and subserve memory, attention, and executive function.^39^^–^^42^ These WM microstructural alterations partially mediated the relationship between ADPRS and cognitive performance, particularly executive function and processing speed. Collectively, our findings suggest that genetic vulnerability to AD manifests in microstructural disorganization of specific WM tracts long before clinical symptoms appear.

Our results extend previous work linking AD genetic risk to brain structure and function. Although several studies have examined the associations between higher ADPRS and reduced hippocampal volume, cortical thinning in AD-vulnerable regions,^26^^,^^27^^,^^43^ or altered WM integrity,^25^^,^^44^ others have failed to detect significant effects using conventional volumetric measures such as total GM, WM, and hippocampus volume.^44^^,^^45^ This inconsistency likely arises from the sensitivity of imaging modalities, the specificity of analytic strategies, and the statistical power. Many earlier studies had modest sample sizes (< 1000 participants)^46^ or focused on global volumetric or cortical thickness measures, which may be too coarse to capture subtle, spatially selective alterations associated with genetic risk. By contrast, our large sample (n > 36000), tract-specific diffusion modeling and inclusion of biologically informed diffusion model provided sufficient sensitivity to capture regionally confined WM microstructural changes associated with ADPRS.

Importantly, the observed WM microstructural patterns, characterized by reduced ICVF and FA coupled with increased MD, AxD, and RD in CGH, Fx, PTR, SFO and TAP, were not widespread but instead spatially selective. This spatial specificity argues against a nonspecific effect of polygenic risk on overall brain integrity and instead supports a model of selective vulnerability in structurally and functionally critical WM tracts. In addition, the reductions in ICVF and alterations in OD observed in regions such as the PTR, Fx, and PCR were consistent with the changes in DTI-derived metrics, suggesting biological alterations such as decreased axonal density and axonal injury in these regions. These regions are consistent with early microstructural vulnerability involving myelin breakdown, axonal loss, or neuroinflammation, which are increasingly recognized as contributors to AD pathophysiology and that are under partial genetic control.^8^^,^^47^^,^^48^ This selective vulnerability pattern aligns with neuropathological staging models of AD and suggests that polygenic risk may manifest preferentially in structurally and functionally critical WM circuits. Rather than inducing global neurodegeneration, cumulative genetic liability appears to target specific tracts such as the Fx, TAP, and PTR, which are closely tied to memory and limbic network integrity and may be more susceptible to early disruption in the pathogenesis of AD.

Furthermore, Fx and PTR, which were consistently affected in our analysis, have been identified in prior work as regions implicated in cognitive decline and vulnerability to AD pathology,^39^^,^^49^ lending further biological plausibility to our results. Additionally, several of the affected tracts in our study, including the CGH, Fx, and SFO, are known to be not only vulnerable to age-related degeneration and have been implicated in network-based models of AD progression.^50^ As such, our data support the notion that polygenic risk contributes to microstructural alterations in critical WM tracts long before clinical symptoms become apparent.

From a cognitive perspective, our findings help to bridge the gap between genetic risk and functional outcomes. We found that higher ADPRS was modestly yet significantly associated with poorer performance in executive function, episodic memory, and processing speed, with the most pronounced effects observed for TMT-B (executive control), PAL (verbal memory), and SDS (complex processing speed). Notably, these tasks are known to depend heavily on long-range WM connectivity, particularly within fronto-thalamic and temporo-parietal circuits.^51^^,^^52^ Our mediation analysis further demonstrated that WM alterations, particularly reduced FA and increased RD in the PTR, partially mediated the relationship between ADPRS and performance on tasks such as TMT-B and SDS, underscoring the importance of WM as an early substrate in AD-related cognitive impairment. Although indirect effects accounted for a small proportion of the total variance (~4.48%), these findings align with previous reports of WM involvement in AD risk,^7^^,^^13^ and support the interpretation that microstructural degeneration is a plausible intermediate pathway linking genetic risk to cognitive decline. Importantly, few previous investigations have explored the mediation pathway from ADPRS to cognition via tract-specific WM metrics derived from biologically informed models such as NODDI.^24^^,^^37^ Thus, our findings offer novel insight into potential mechanistic routes through which cumulative genetic burden impacts cognition, even in clinically asymptomatic individuals.

These results have important translational implications. Combining PRS with advanced diffusion MRI offers a promising strategy to identify individuals at elevated risk for AD before symptom onset. Furthermore, tract-specific WM signatures could enhance personalized risk prediction models and guide early intervention trials. By focusing on specific tract or network integrity rather than overt atrophy, this framework bridges the gap between genetic predisposition and clinically detectable decline, paving the way toward precision prevention in neurodegenerative disorders.

Nonetheless, several limitations warrant consideration. First, the cross-sectional nature of our analysis precludes inference regarding the temporal dynamics of genetic risk, brain structure, and cognition. Longitudinal data are necessary to establish whether the observed WM changes precede or predict clinical conversion to AD. Second, the PRS captures only common variants and does not account for rare variants, epigenetic modifications, or environmental influences that may modulate risk expression. Third, the present study relied on ADPRS as an indirect proxy for AD related pathology and did not include direct pathological biomarkers, such as amyloid or tau PET imaging, or cerebrospinal fluid/plasma biomarkers. As a result, we cannot directly confirm whether the observed WM microstructural alterations reflect underlying AD pathology at the individual level. Fourth, vascular risk factors such as hypertension, diabetes, and smoking history, as well as WM hyperintensity burden, were not included as covariates in the primary analyses. These factors are known to influence WM microstructure and cognitive function and may interact with neurodegenerative processes.^53^ Fifth, the UKB cohort consists mainly of individuals of European ancestry, limiting generalizability to other populations. Finally, although NODDI provides enhanced biological specificity, it still relies on model assumptions and is sensitive to signal-to-noise limitations. Future studies integrating multimodal data, including transcriptomics, plasma biomarkers, and lifestyle measures, as well as detailed vascular risk profiles and quantitative white matter hyperintensities measures, will be essential to clarify mechanistic pathways underlying early WM vulnerability in AD.

## Conclusion

Our study provides robust evidence that ADRPS is associated with selective microstructural changes in WM and subtle cognitive inefficiencies in clinically asymptomatic individuals. These findings underscore the value of combining genomic and neuroimaging data to characterize early neural consequences of genetic risk and inform preventative strategies. By focusing on tract-level vulnerability rather than global atrophy, and on probabilistic risk rather than deterministic pathology, this approach offers a foundation for precision monitoring and intervention in the earliest stages of AD.

## Figures and Tables

**Table S1:**
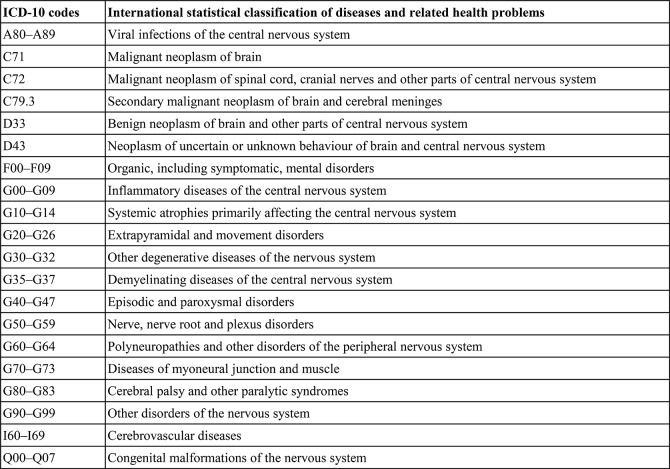
ICD-10 codes applied for exclusion in this study

**Table S2:**
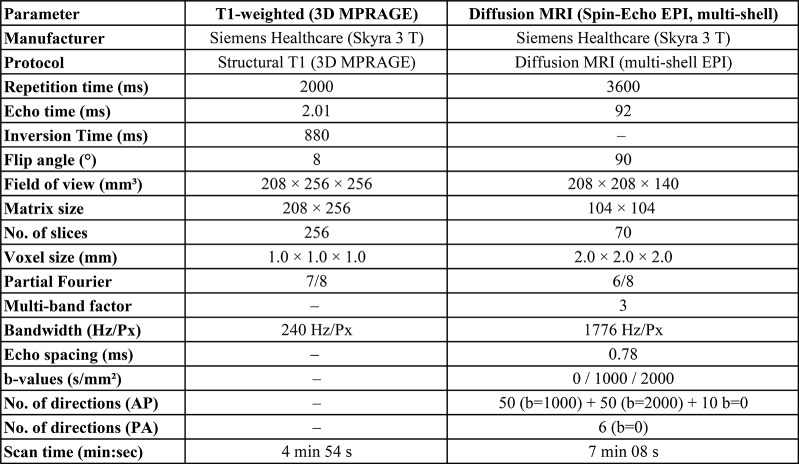
MRI acquisition parameters for the T1WI and dMRI sequence

**Table S3:**
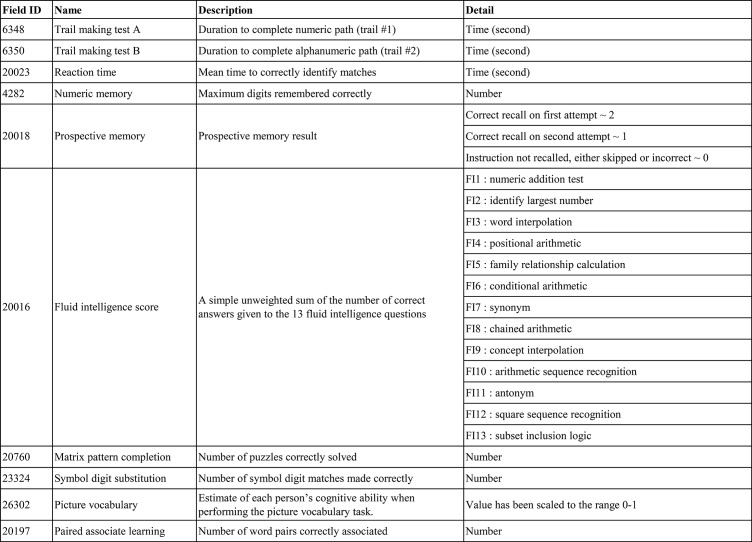
Description of the cognitive test and their scoring metrics used in this study

**Table S4:**
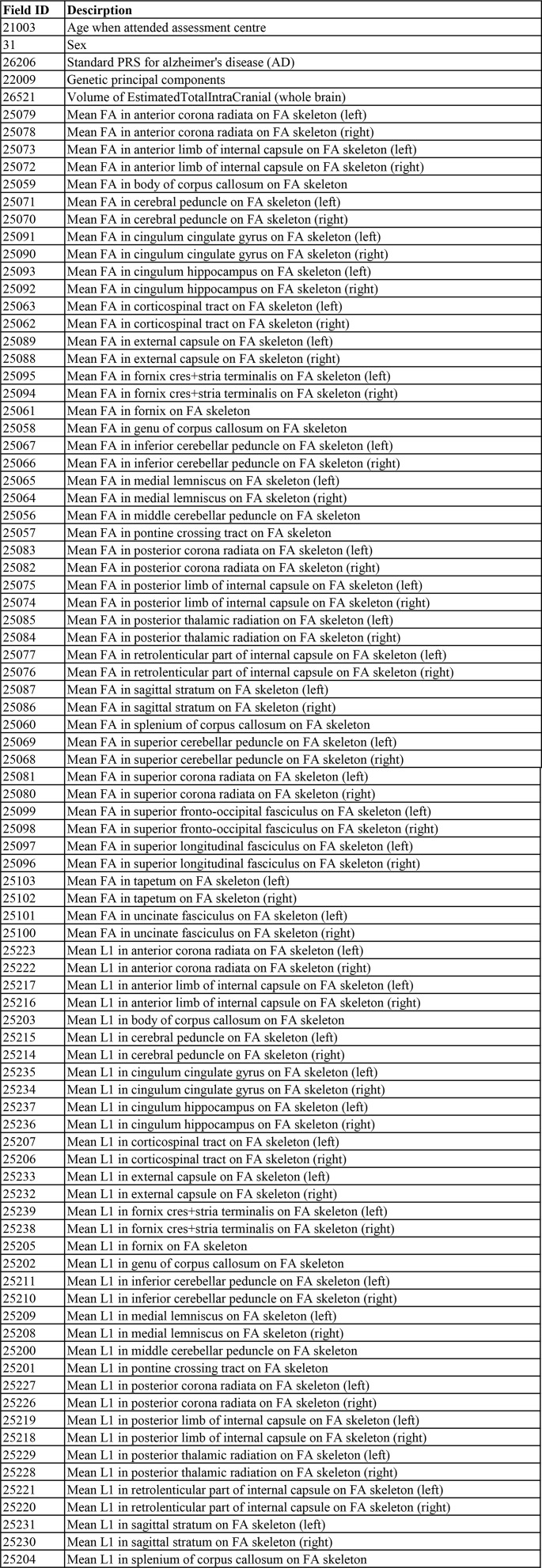
The specific imaging, ADPRS, and cognitive variables used in this study

**Table:**
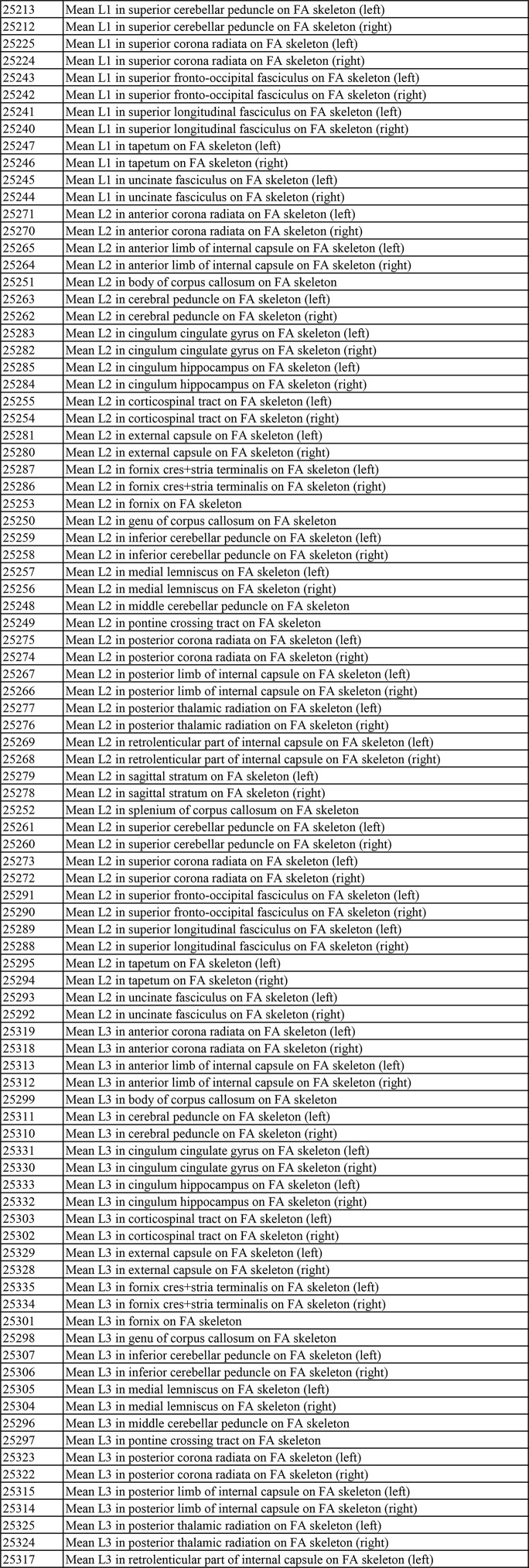


**Table:**
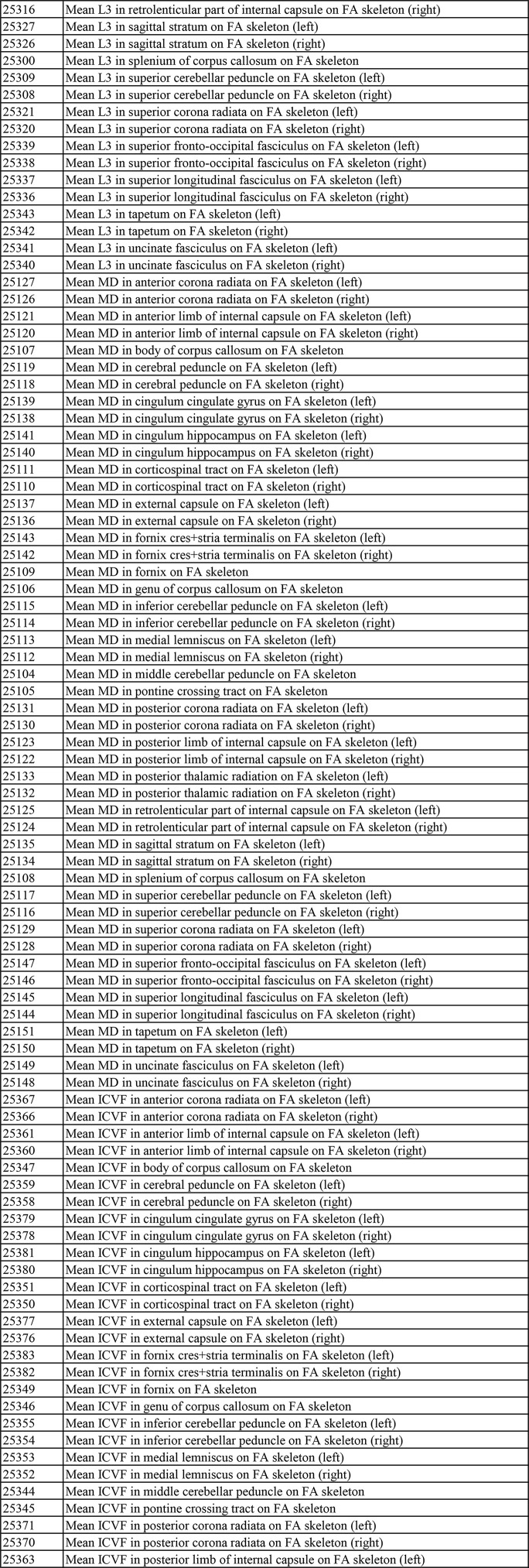


**Table:**
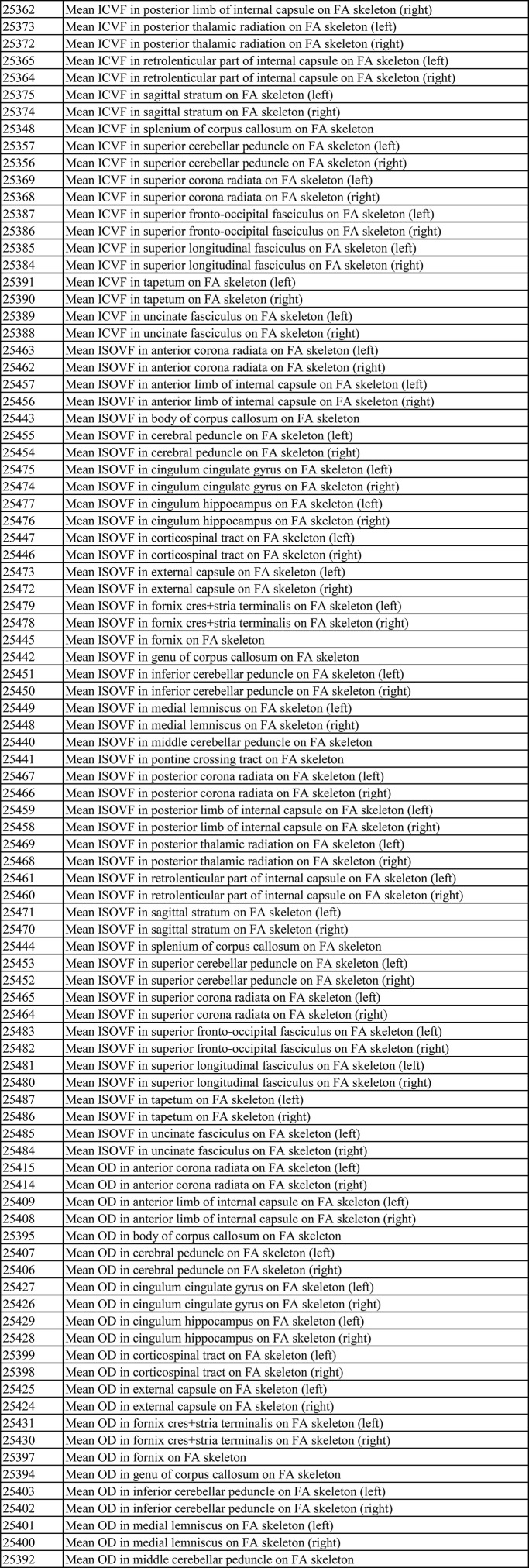


**Table:**
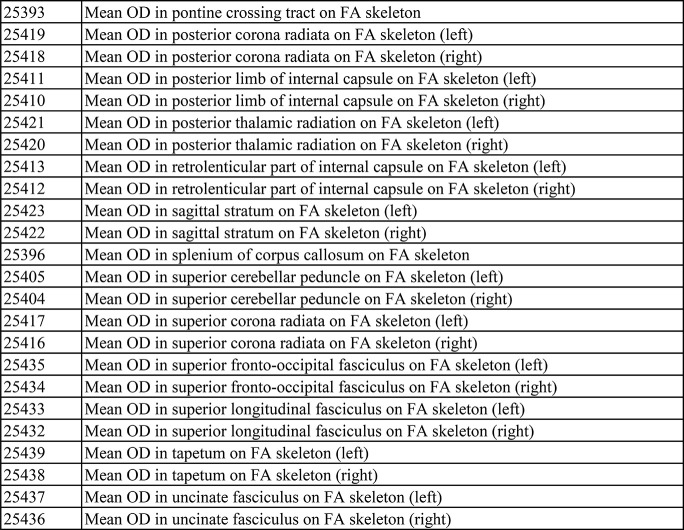


**Table S5:**
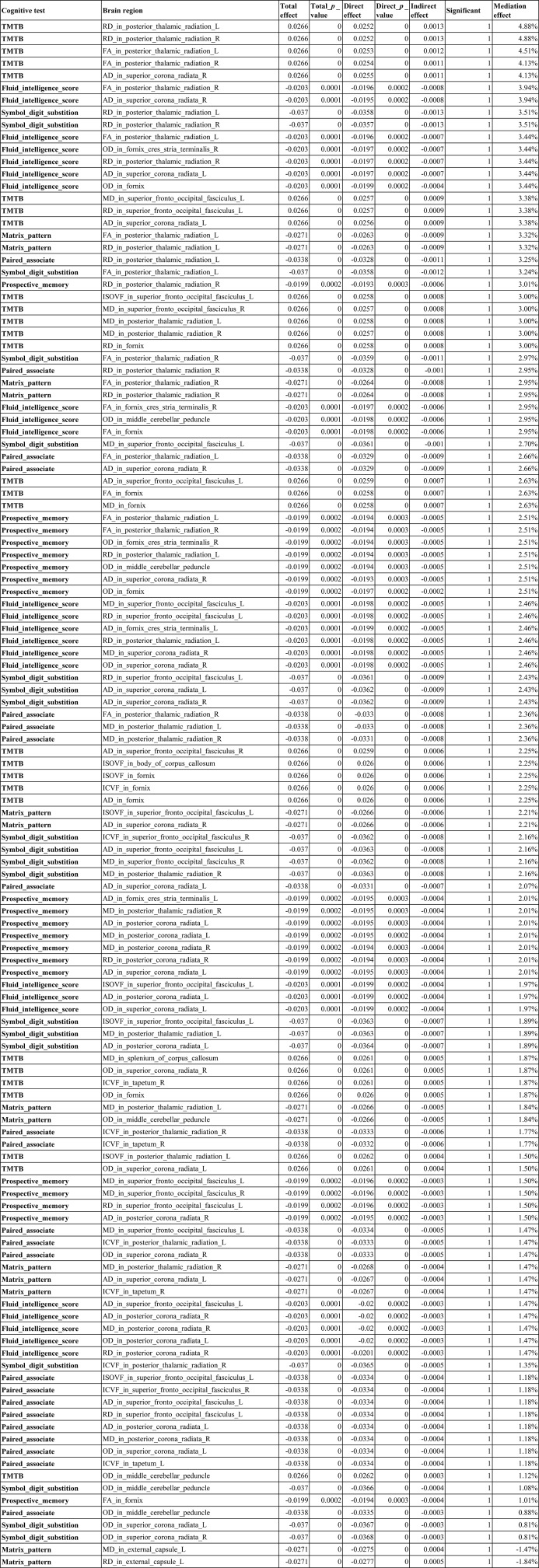
Full results from all mediation models in this study

**Table 1:** Demographics and clinical characteristics of participants in this study

Variable	Total No.	Overall		ADPRS				*P* value		
				Q1	Q2	Q3	Q4			
ADPRS (mean ± SD)	36400	0.032 ± 0.99		− 1.10 ± 0.36	− 0.37 ± 0.16	0.22 ± 0.20	1.38 ± 0.64		**0.0000**	
Age (mean ± SD)	36400		64.06 ± 7.72	64.19 ± 7.76	64.23 ± 7.84	64.05 ± 7.61	63.79 ± 7.65		**0.0001**	
Female No. (%)	36400		19317 (53.07%)	4879 (53.62%)	4773 (52.45%)	4831 (53.09%)	4834 (53.12%)		0.7178	
ICV (mean ± SD)	35952		1546437.87 ± 153648.08	1544002.56 ± 154431.66	1551026.32 ± 158801.51	1545491.58 ± 149769.55	1545233.02 ± 151370.60		0.7988	
TMT-A (mean ± SD)	25850		226.80 ± 86.24	226.82 ± 85.17	225.81 ± 86.83	226.53 ± 83.46	228.04 ± 89.45		0.3631	
TMT-B (mean ± SD)	25260		571.30 ± 254.48	568.75 ± 253.26	567.15 ± 251.53	571.80 ± 250.41	577.56 ± 262.56		**0.0306**	
RT (mean ± SD)	34025		595.93 ± 109.62	597.39 ± 112.67	596.01 ± 110.13	593.60 ± 106.56	596.69 ± 109.00		0.3966	
NM (mean ± SD)	26332		6.76 ± 1.27	6.75 ± 1.25	6.81 ± 1.28	6.76 ± 1.29	6.74 ± 1.26		0.3012	
PM (mean ± SD)	34216		1.79 ± 0.52	1.79 ± 0.51	1.79 ± 0.51	1.79 ± 0.52	1.78 ± 0.52		0.0578	
PAL (mean ± SD)	26144		6.90 ± 2.64	7.00 ± 2.62	6.95 ± 2.58	6.84 ± 2.67	6.82 ± 2.69		**0.0000**	
FIS (mean ± SD)	33688		6.63 ± 2.05	6.66 ± 2.06	6.67 ± 2.04	6.61 ± 2.06	6.58 ± 2.02		**0.0050**	
SDS (mean ± SD)	25890		18.97 ± 5.27	19.12 ± 5.22	19.01 ± 5.19	18.96 ± 5.33	18.79 ± 5.34		**0.0003**	
MPC (mean ± SD)	25878		8.01 ± 2.13	8.06 ± 2.15	8.03 ± 2.11	8.02 ± 2.16	7.95 ± 2.09		**0.0057**	
PV (mean ± SD)	23844		0.40 ± 0.08	0.40 ± 0.08	0.40 ± 0.08	0.40 ± 0.08	0.40 ± 0.08		0.0701	

Statistically significant *P* values (< 0.05) are shown in bold.ADPRS, polygenic risk score of Alzheimer’s disease; FIS, fluid intelligence score; ICV, intracranial volume; MPC, matrix pattern completion; NM, numeric memory; PAL, paired associate learning; PM, prospective memory; PV, picture vocabulary; RT, reaction time; SDS, symbol digit substitution; SD, standard deviation; TMT-A, trail making test A; TMT-B, trail making test B.

**Table 2:** Association between ADPRS and cognitive performance

Cognitive tests	*β* coefficient	SE	*P* value	*P* value (FDR adjusted)
TMT-A	0.0128	0.0059	**0.0298**	**0.0426**
TMT-B	0.0266	0.0059	**0.0000**	**0.0000**
RT	0.0045	0.0052	0.3787	0.3787
SDS	− 0.0370	0.0056	**0.0000**	**0.0000**
PM	− 0.0199	0.0053	**0.0002**	**0.0003**
PV	− 0.0076	0.0063	0.2304	0.2560
PAL	− 0.0338	0.0060	**0.0000**	**0.0000**
NM	− 0.0117	0.0061	0.0546	0.0682
MPC	− 0.0271	0.0059	**0.0000**	**0.0000**
FIS	− 0.0203	0.0053	**0.0001**	**0.0003**

Statistically significant *P* values (< 0.05) are shown in bold.FDR, false discovery rate; FIS, fluid intelligence score; MPC, matrix pattern completion; NM, numeric memory; PAL, paired associate learning; PM, prospective memory; PV, picture vocabulary; RT, reaction time; SDS, symbol digit substitution; SE, standard error; TMT-A, trail making test A; TMT-B, trail making test B.

## Supplementary Materials

Supplementary figures are available online.
